# Health Economic Evaluations of Cancer in Brazil: A Systematic Review

**DOI:** 10.3389/fpubh.2018.00205

**Published:** 2018-07-27

**Authors:** Alessandro G. Campolina, Tania Y. Yuba, Tassia C. Decimoni, Roseli Leandro, Maria del Pilar Estevez Diz, Hillegonda M. D. Novaes, Patrícia C. de Soárez

**Affiliations:** ^1^Department of Oncology, Instituto do Câncer do Estado de São Paulo, São Paulo, Brazil; ^2^Instituto do Câncer do Estado de São Paulo, São Paulo, Brazil; ^3^National Institute for Science and Technology for Health Technology Assessment (IATS/CNPq), Porto Alegre, Brazil; ^4^Preventive Medicine, Faculdade de Medicina, Universidade de São Paulo, São Paulo, Brazil

**Keywords:** costs and cost analysis, cost-benefit analysis, health care costs, neoplasms, Brazil

## Abstract

**Background:** A large number of health economic evaluation (HEE) studies have been published in developed countries. However, Brazilian HEE literature in oncology has not been studied. Objective: To investigate whether the scientific literature has provided a set of HEE in oncology capable of supporting decision making in the Brazilian context.

**Methods:** A systematic review was conducted to identify and characterize studies in this field. We searched multiple databases selecting partial and full HEE studies in oncology (1998-2013).

**Results:** Fifty-five articles were reviewed, of these, 33 (60%) were full health economic evaluations. Type of cancers most frequently studied were: breast (38.2%), cervical (14.6%), lung (10.9%) and colorectal (9.1%). Procedures (47.3%) were the technologies most frequently evaluated. In terms of the intended purposes of the technologies, most (63.6%) were treatments. The majority of the incremental cost-effectiveness ratios (ICERs) reported have been below the cost-effectiveness threshold suggested by the World Health Organization (WHO).

**Conclusions:** There has been an increase in the number of HEEs related to cancer in Brazil. These studies may support decision-making processes regarding the coverage of and reimbursement of healthcare technologies for cancer treatment in Brazil.

## Introduction

Every year, there are approximately 12.7 million new cases of cancer worldwide. It is estimated that, by 2030, the annual number of new cases of cancer in Latin America and the Caribbean will reach 1.7 million, resulting in more than 1 million deaths (1).

In Brazil, the annual number of new cases of cancer is expected to reach 600,000 by the end of 2018. Among males, the most common types of neoplasia are cancer of the prostate, lung, stomach, and oral cavity, whereas the most common types among women are cancer of the breast, cervix, lung, and thyroid gland, as well as nonmelanoma skin cancer (2). Within this context, major advances in the early diagnosis of certain types of cancer and greater understanding of the pathogenesis of neoplasia have led to the development of strategies for preventing and reducing the risk of cancer. Such advances, together with the development of new therapies, have helped reduce the rates of cancer-related mortality in various countries. However, this success has resulted in a substantial increase in health care expenditures for cancer treatment (3).

In parallel with the advances in cancer management, health care systems in Latin America have faced challenges related to demographic and epidemiological changes, characterized by the increased incidence of chronic noncommunicable diseases (1). Despite much progress being made in the restructuring of health care systems, such as: progress on development of cancer registries, adjustments to funding toward universal health care and support of the underserved, there are still a number of obstacles to the efficient management of chronic diseases. In the case of cancer, one particular challenge is to offer a basic level of care, including initiation of programmes for primary cancer prevention (1).

In this scenario, health economic evaluations (HEEs) play a fundamental role by helping identify the best means of allocating the financial resources of a health care system to provide the maximum benefit to the population served (4). There are two major types of HEEs: partial and full (5). Partial HEEs describes the costs and consequences of a single service or program, or compare two or more interventions only in terms of their costs. Full HEE designs include cost-consequences analysis, cost-minimization analysis, cost-effectiveness analysis, cost-utility analysis, and cost-benefit analysis (5).

A large number of HEE studies in oncology have been published in developed countries and their results highlight many opportunities for efficient investment in cancer care across cancer sites and levels of prevention (6, 7). The characteristics of the body of HEEs related to cancer in Brazil remain unknown. A systematic review of these studies could identify studies that were or are relevant for informing health care priorities and their potential economic impact (8).

In addition, the information collected may contribute to aid decision making at different levels of the health system. Clinicians would be able to better visualize the high cost of some prescriptions and the magnitude of the benefits related to them, contributing to a more rational and responsible use of the technologies offered by the health system. Researchers would be able to visualize knowledge gaps to propose health technology assessment not yet carried out in the Brazilian context. Healthcare managers would benefit from a comparative panel of the various cancer technologies, previously evaluated in Brazil. Policymakers would be able to visualize the most efficient technologies in proposing strategies and programs for delivering cancer care in the country.

Therefore, the research question that moves the present study is to investigate whether the scientific literature has provided a set of HEE in oncology capable of supporting decision making in the Brazilian context. The objective of this study was to describe the scientific literature of Brazil, in terms of HEEs related to cancer, over an extended period (1998-2013) (8).

## Methods

This systematic review forms part of a larger research project that systematically reviewed all HEEs related to Brazil and published between 1980 and 2013 (9). This study is in accordance with the guidelines for systematic review of HEEs published by the UK National Health Service (NHS) Center for Reviews and Dissemination (10). We searched multiple databases, including Medline; *ExcerptaMedica*; the Latin American and Caribbean Health Sciences Literature database; the Scientific Electronic Library Online; the database of the Center for Reviews and Dissemination; the NHS Economic Evaluation Database; the NHS Health Technology Assessment database; and Health Economics database of the Brazilian Virtual Library of Health (search strategy in Appendix 1 in the Supplementary Material). We searched the citation indexes: Scopus; Web of Science; and the Brazilian Network for the Evaluation of Health Technologies. We also performed hand-searching, a way to scan the content of journals and other sources, page by page. It was done to identify articles from journals that are not indexed by electronic database and for full identification of relevant reports published in journals, even for those that are indexed in Medline (10). We performed hand-searching from the reference lists of included articles, and all issues of the Brazilian Journal of Health Economics (BJHE), a non-indexed journal in that period.

Articles were included if they were partial or full economic evaluations, if they dealt with cancer, if they were conducted in Brazilian setting, and if at least one of the authors was affiliated with an institution in Brazil (5). The language of article was not an exclusion criterion. For the inclusion of economic evaluation studies, the main outcomes considered, in addition to the cost of illness, were: life year gained (LYG), deaths averted, diagnostics averted, quality-adjusted life years (QALYs) and disability-adjusted life years (DALYs).

QALYs and DALYs are technically similar in that they both express health in time (life years) and give a weight to years lived with a disease. However, the difference between these measures depends on whether the quality of life is expressed as a gain (QALY) or a loss (DALY) (11). Since DALY is a widely used measure in economic evaluations conducted in developing countries or in a country for which no local health state preference values exist (5), the World Health Organization (WHO) have recommended their use in cost-effectiveness analysis and proposed a willingness to pay threshold of one to three times the per capita gross domestic product (GDP) per DALY averted, which have been considered an alternative to the QALY gained as a measure of health benefit (12). So, for the purpose of the present study the QALY gained will be used as a proxy of DALY averted, when using the WHO threshold.

Two reviewers, working independently, selected studies and extracted data using a template developed specifically for this study. Data extracted from each study included the following: year and journal of publication; type of HEE (partial and full); category of technology assessed (medications, vaccines, equipment, procedures, public health, and health promotion programs); purpose of the technology assessed (treatment, prevention, screening and diagnosis); the type of affiliation of the first author; the geographical location of the first author; conflicts of interest, as defined estimates of cost-effectiveness; and the conclusions of the studies (favorable, unfavorable, or neutral) (11). To compare the results of the studies, we converted the incremental cost-effectiveness ratios (ICERs) into United States Dollars (USD) for 2013. For studies in which the cost year was not specified, we assumed that the cost year was the same the year of publication, a strategy that has been adopted in previous reviews of the literature (13). To transform every monetary value at the same year to compare, we used the Brazilian Extended National Consumer Price Index (IPCA), available from the Central Bank of Brazil^1^ As an indicator of cost-effectiveness, we used the WHO cost-effectiveness threshold (12). A qualitative narrative synthesis was conducted.

## Results

In total 11,841 records were identified from database searches, and 105 further articles were identified in other sources (BJHE - Brazilian Journal of Health Economics, SISREBRATS-Brazilian Network for Health Technology Assessment Database, and gray literature). We identified 9,304 non-duplicate citations, of which 721 were recognized as potentially relevant and full papers were retrieved. Out of the 721 studies 186 of them were excluded. Reasons for exclusion included: thesis (50 studies), not HEE (88 studies), no Brazilian author (19 studies), reviews (98), and other (11 studies). It resulted in 535 HEEs studies (9).

**Figure 1:**
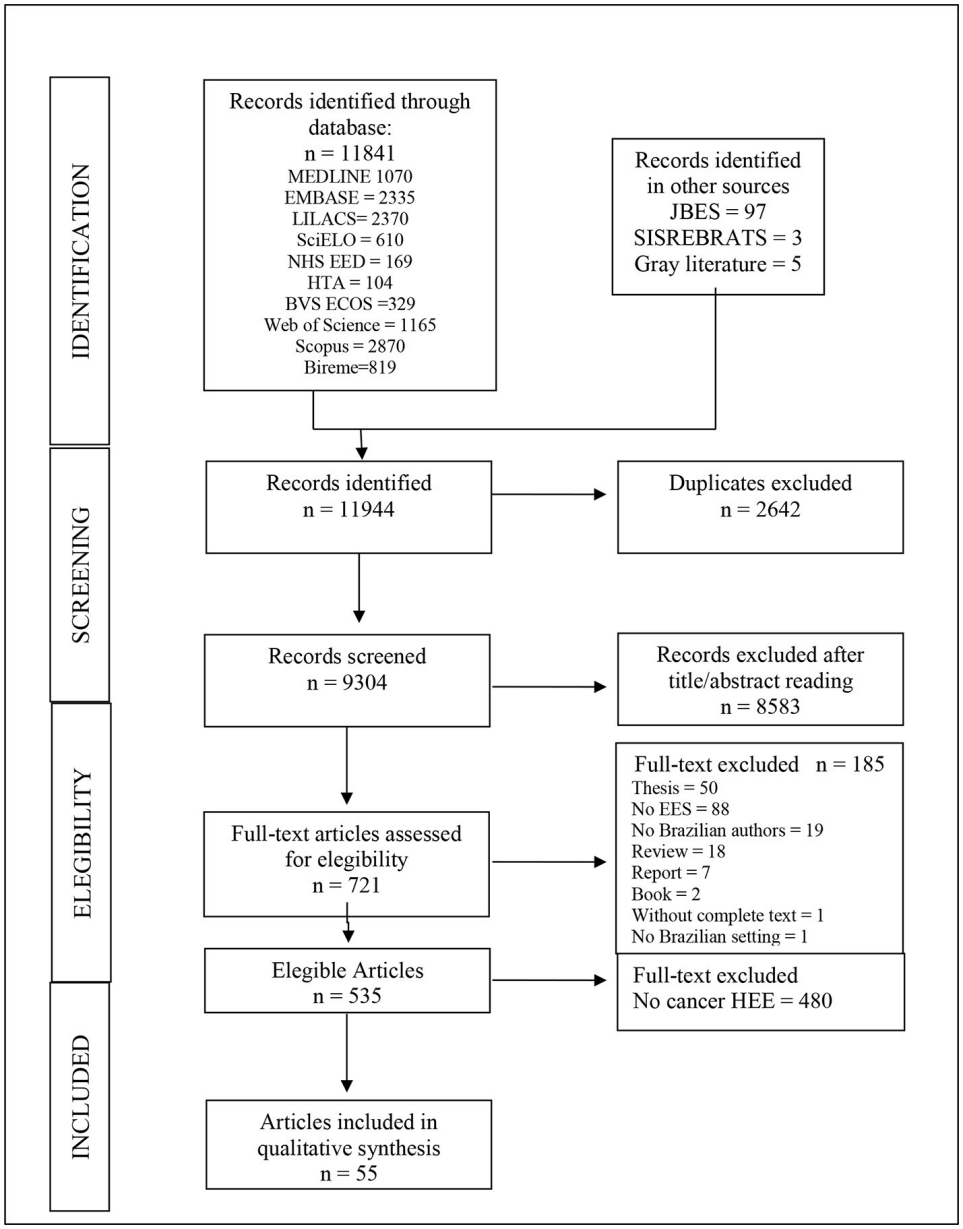
Flow diagram of the process for the selection of health economic evaluations related to cancer in Brazil, 1998–2013. Bireme, Biblioteca Regional de Medicina (Regional Library of Medicine); BVS ECOS, Biblioteca Virtual em Saúde Economia da Saúde (Health Economics [database] of the [Brazilian] Virtual Library of Health); EED, Economic Evaluation Database; EMBASE, Excerpta Medica; JBES, Jornal Brasileiro de Economia da Saúde (Brazilian Journal of Health Economics); LILACS, Latin American and Caribbean Health Sciences Literature; SciELO, Scientific Electronic Library Online; SISREBRATS, Sistema de Informação da Rede Brasileira de Avaliação de Tecnologias em Saúde (Brazilian Network for the Evaluation of Health Technologies).

**Figure 2:**
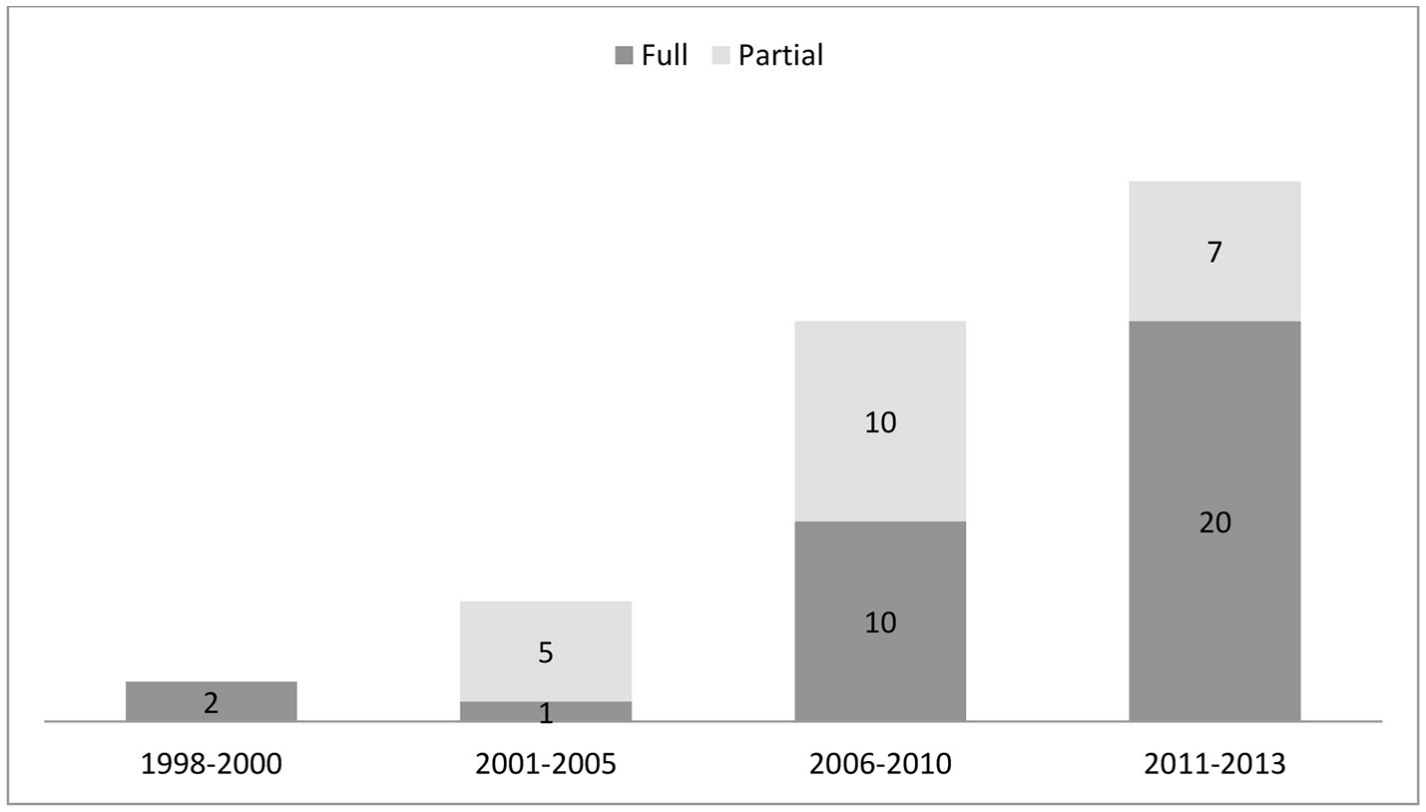
Number and proportional distribution of health economic evaluations related to cancer in Brazil, by publication date and type, 1998–2013.

Among those 535 HEEs studies related to Brazil Figure 1, there were 55, published between 1998 and 2013, that dealt with cancer-related technologies (12, 14–68). The yearly number of studies increased over the period as did the proportion of full HEEs (in relation to that of partial HEEs) Figure 2. Of the 55 cancer-related HEEs, 47 (85.5%) were published between 2006 and 2013 and 33 (60.0%) were full HEEs. As shown in the Table 1, the most frequent study design was cost-effectiveness analysis (23.6%) and the least frequent cost-minimization analysis (3.6%). Among the partial HEEs, the most frequent study design was cost-description (14.6%), followed by cost-of-illness (10.9%).

**Table 1:** Characteristics of health economic evaluations related to cancer in Brazil, 1998-2013.

**Characteristic**	***n***	**%**
**TYPE OF HEE**		
Full	33	60.0
Cost-effectiveness analysis	13	23.6
Cost-utility analysis^*^	11	20.0
Cost-consequences analysis	7	12.7
Cost-minimization analysis	2	3.6
Partial	22	40.0
Cost-description study^†^	8	14.6
Cost analysis	8	14.6
Cost-of-illness study	6	10.9
**TYPE OF TECHNOLOGY EVALUATED**		
Procedures	26	47.3
Medications	20	36.4
Vaccines	3	5.4
Equipment	2	3.6
Public health/health promotion programs	2	3.6
Combined modalities^§^	2	3.6
**TYPE OF CANCER ADDRESSED**		
Breast cancer	21	38.2
Cervical cancer	8	14.6
Lung cancer	6	10.9
Colorectal cancer	5	9.1
Prostate cancer	3	5.4
Skin cancer	2	3.6
Other^‡^	10	18.2
**REGION OF AFFILIATION (FIRST AUTHOR)**		
Southeast	45	81.8
South	4	7.3
Northeast	2	3.6
Central-west	2	3.6
North	2	3.6
**TYPE OF AFFILIATION (FIRST AUTHOR)**		
Academia	22	40.0
Health care facility	18	32.7
Consultancy	7	12.7
Industry	3	5.4
Public administration	4	7.3
Research institute	1	1.8
**JOURNAL OF PUBLICATION**		
Indexed	46	83.6
Not indexed	9	16.4
**CONFLICT OF INTEREST**		
No	38	69.1
Yes	17	14.6

* *Six of these conducted cost-utility analysis and cost-effectiveness analysis simultaneously*.

† *One of these was a budget impact analysis*.

‡ *Cancer in general (1.8%); head and neck cancer (1.8%); esophageal cancer, lung cancer, or laryngeal cancer (3.6%); chronic myeloid leukemia (1.8%); kidney cancer (1.8%); cancer of the oral cavity or pharyngeal cancer (1.8%); bone metastasis (1.8%); febrile neutropenia (1.8%); and Kaposi's sarcoma (1.8%)*.

§ *More than one type of technology (medication or procedure)*.

The top five types of cancer addressed in the HEEs were: breast cancer (38.2%); cervical cancer (14.6%); lung cancer (10.9%); colorectal cancer (9.1%); and prostate cancer (5.4%).

As shown in Table 1, the technological modalities most often evaluated in the HEEs identified were procedures (47.3%), followed by medications (36.4%) and vaccines (5.4%). In terms of the intended purposes of the technologies evaluated, most (63.6%) were treatments, whereas screening methods, diagnostic procedures, and preventive measures accounted for 16.4, 14.6, and 5.4%, respectively.

Among the HEEs dealing with breast cancer (14–34), the technologies evaluated included strategies for screening with conventional and digital mammography, as well as with magnetic resonance imaging; a gene expression panel to inform decisions regarding therapy; medications (docetaxel, vinorelbine, trastuzumab, anastrozole, lapatinib, and capecitabine); screening tests for metastasis (mammography, bone scintigraphy, routine chest X-ray, and ultrasound of the liver); and procedures such as postmastectomy breast reconstruction and core biopsy. The HEEs dealing with cervical cancer (35–42) evaluated strategies for screening with liquid-based cytology; human papillomavirus (HPV) detection by hybrid capture assay; Pap smear; molecular biology methods for the diagnosis of HPV infection; and vaccination against HPV. Among the HEEs related to lung cancer (43–48), the technologies evaluated included medications such as erlotinib, gefitinib, and the pemetrexed-cisplatin combination; and strategies for clinical follow-up and metabolic staging with positron emission tomography. The HEEs related to colorectal cancer (49–53) dealt with technologies such as chemotherapy regimens (modified 5-fluorouracil/leucovorin/oxaliplatin; 5-fluorouracil/folinic acid/oxaliplatin; folinic acid/5-fluorouracil/irinotecan; capecitabine/oxaliplatin; and capecitabine/irinotecan); and a procedure involving transanal endoscopic microsurgery.

Analyzing the geographic distribution of the affiliations of the first authors, we noted a concentration of HEEs from southeastern Brazil, which accounted for 45 (81.8%) of the 55 studies identified Table 1, of which 31 (56.4%) and 12 (21.8%) were from the states of São Paulo and Rio de Janeiro, respectively, both of which are in the southeastern region. The first authors of the selected articles had the following types of affiliations Table 1: academia, in 22 studies (40.0%); health care facilities, in 18 (32.7%); and industry or consultancy, in 10 (18.2%). Potential conflicts of interest were identified in 14.6% Table 1. The majority (83.6%) of the HEEs evaluated were published in indexed journals Table 1.

The ICERs were presented in relation to two outcome measures: LYG and QALYs gained. The ICER per QALY gained ranged from USD466.45 to USD374,630.96.

Of the 55 studies evaluated in our review, 23 (41.8%) calculated the ICERs. We found that the ICERs for technologies used in the treatment of breast cancer ranged from USD914.89 to USD285,874 (across 8 studies), compared with USD436.48 to USD16,706.75 (across 6 studies) for cervical cancer, USD6,380 to USD320,880 (between 2 studies) for lung cancer, USD47,399 to USD66,892 (between 2 studies) for colorectal cancer, and USD3,123 to USD190,193 (across 4 studies) for other types of cancer.

**Figure 3:**
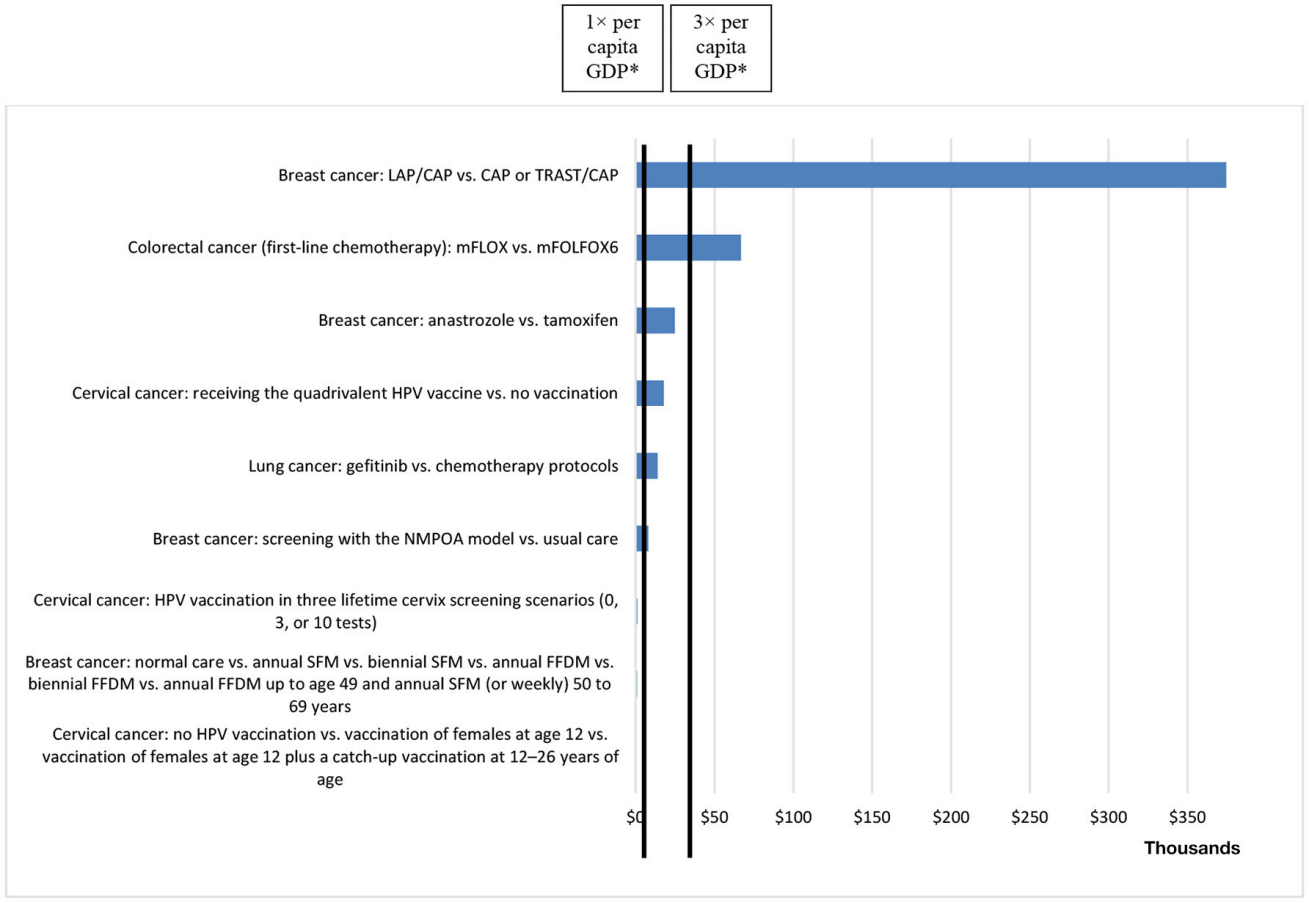
ICERs per QALY gained, in United States Dollars (at 2013 rates), reported in health economic evaluations related to cancer in Brazil, 1998–2013 (× USD 1,000). *1× per capita GDP = USD 14,588.62; 3× per capita GDP = USD 43,765.86 (2013). CAP, capecitabine; LAP, lapatinib; mFLOX, modified 5-fluorouracil, leucovorin, and oxaliplatin; mFOLFOX6, modified 5-fluorouracil, folinic acid, and oxaliplatin (mFOLFOX6); NMPOA, Núcleo Mama Porto Alegre (Breast Center of Porto Alegre); SFM, screen-film mammography; FFDM, full-field digital mammography; TRAST, trastuzumab.

**Figure 4:**
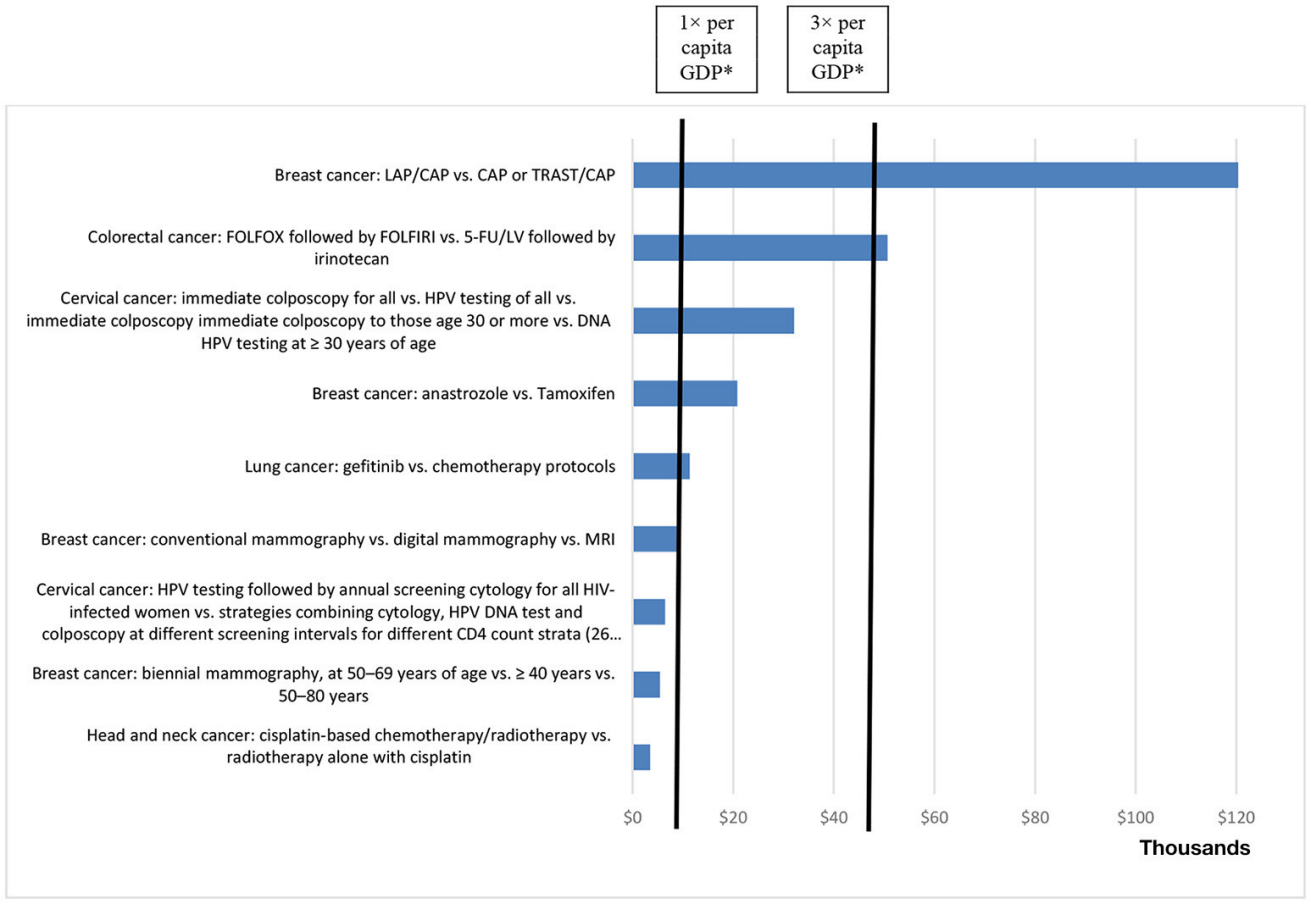
ICERs per life year gained, in United States Dollars (at 2013 rates), reported in health economic evaluations related to cancer in Brazil, 1998–2013 (× USD 1,000). *1× per capita GDP = USD 14,588.62; 3× per capita GDP = USD 43,765.86 (2013). 5-FU/LV, 5-fluorouracil plus leucovorin; FOLFIRI, folinic acid, 5-fluorouracil, and irinotecan; FOLFOX, 5-fluorouracil, folinic acid, and oxaliplatin; MRI, magnetic resonance imaging.

The lowest ICER per QALY value was for vaccination against HPV (vs. no vaccination), whereas the highest value was for the treatment of breast cancer (lapatinib + capecitabine vs. capecitabine or trastuzumab + capecitabine), as depicted in Figure 3. The ICER per LYG ranged from USD3,505.45 to USD120,832.12. The lowest ICER per LYG value was for the treatment of head and neck cancer (cisplatin-based chemotherapy vs. radiotherapy), whereas the highest value was for the treatment of breast cancer (lapatinib + capecitabine vs. capecitabine or trastuzumab + capecitabine), as depicted in Figure 4. In only four of the HEEs evaluated (19,57,58,67), the ICERs reported were above the threshold of three times the per capita GDP per DALY averted, established by the WHO (12).

## Discussion

Because of the shortage of health care resources, together with the great technological development over recent decades, health technology assessments (HTAs), like HEEs, have come to play a greater role in the process of incorporating new technologies into health care systems (5). In Brazil, the growth of scientific literature in this field of knowledge has occurred within the context of the process of devising the Brazilian Ministry of Health (MoH) statement of the National Policy on Health Technologies Management. In the second half of the preceding decade, there was a significant increase in the number of HEEs related to cancer in Brazil, most of which were published in indexed journals (4).

There was a predominance of HEEs evaluating procedures and medications, which can be attributed to the fact that, in Brazil, the articulation of the *Agência Nacional de Vigilância Sanitária* (ANVISA, National Health Surveillance Agency) with MoH Department of Science, Technology, and Strategic Resources and with the National Health Agency. Since 2000, ANVISA has been regulating the sales of medications. In 2004, ANVISA required HEE as part of the decision-making process related to determining the price of any new medication (4).

In other countries, medications have also been the health technologies most often evaluated in economic studies (7). However, when dealing with a complex condition like cancer, studies evaluating other types of technologies, such as surgical interventions, are of great importance. Without such studies, decisions regarding incorporation of such procedures would be made at the health care facility level, with or without the approval of local committees for the evaluation and management of health technologies (6, 69).

There is a lack of robust evidence on the cost-effectiveness of surgical intervention in the treatment of breast, colorectal, and prostate cancer (69). Among the 17 cancer-related economic evaluations identified in a review, surgical interventions for the treatment of breast cancer were evaluated in only three, all of which were published before 2003. Because surgical interventions are often associated with great potential benefits (cure vs. no cure) or harms (morbidity and mortality), the limited number of economic evaluations of such interventions raises the question of what type of evidence has been used in the development of the corresponding coverage and reimbursement recommendations (7, 69).

Although evidence on the cost effectiveness of radiotherapy in cancer is heterogeneous, a systematic review highlights the importance of the identification of specific types of patients in whom radiotherapy is cost effective (70). Furthermore, it is important to consider that studies might be dated due to improvements in clinical practice, since radiotherapy techniques have evolved rapidly over the last decade and many newer techniques are currently being evaluated in clinical trials (7, 70).

The development of targeted therapies and personalized cancer medicine appears to be another key issue (71). The consequences for technology joint assessment of a diagnostic technology and a treatment has additional methodological complexities and are not yet fully worked out. One view is that the basic principles of HTA still apply and that this is not different from undertaking HTA for diagnostics and treatments in other diseases. Another view is that the new paradigm changes not only the regulatory assessments but also the way the broader aspects of the technology are assessed (71).

In the present review, we found that breast cancer was the type of neoplasia most often addressed in economic evaluations related to Brazil. We identified two HEEs related to the use of mammography in breast cancer screening in Brazil. Despite the fact that the scientific literature has contained evidence that such screening reduces mortality since 1993 (1), those two studies (a partial HEE and a full HEE) were published only in 2005 and 2010, respectively. Similarly, the breast cancer drugs anastrozole and trastuzumab, approved by the United States Food and Drug Administration (FDA) in 1996 and 1998, respectively, were not evaluated in HEEs related to Brazil until 2009 and 2008, respectively (27, 33). Although HEEs related to breast cancer have been conducted only recently in Brazil, as have those related to other aspects of oncology, these results indicate the importance of breast cancer, not to mention that of nonmelanoma skin cancer, the former representing the most common type of neoplasia among women in developed and developing countries alike, as well as being the leading cause of cancer death among women worldwide (2).

The second most common topic addressed in HEEs dealing with cancer in Brazil is cancer of the cervix, the importance of which is recognized due to the fact that it is a major public health problem, principally in the northern region of the country, where it has an incidence rate higher than that of any other type of neoplasia (1, 2). Economic studies of HPV screening in the population of Brazil were published in 2006 and 2007, coinciding with FDA approval of the first HPV vaccine, in 2006. The HPV vaccine shows great promise as a tool in the battle against cervical cancer. Its incorporation into the standard practices of the *Sistema Único de Saúde* (SUS, the Brazilian National Health System), via which it has been available since 2014, was supported by HEEs conducted in Brazil (39).

The third most common type of cancer evaluated in HEEs related to cancer in Brazil is lung cancer (2). That apparently reflects the level of concern regarding a condition that is recognized as the leading cause of cancer death worldwide (2, 3). Economic evaluations of lung cancer drugs, commissioned by the pharmaceutical industry, began to appear in Brazil between 2008 and 2012, a period during which the FDA was in the process of approving the anaplastic lymphoma kinase inhibitor crizotinib for the treatment of small cell lung cancer, providing prospects for improved survival (43, 48). Lung cancer is typically detected in the advanced stages, often requiring chemotherapy, despite the high mortality associated with the disease (2). Therefore, it is highly advisable to conduct rigorous economic evaluations of the therapeutic measures employed in cases of lung cancer (6, 7).

Colorectal cancer, which is estimated to be the second and third leading cause of cancer death among women and men, respectively, worldwide, is another important topic to be addressed in HEEs (1–3). The increased incidence of colorectal cancer has been attributed to greater urbanization and to the growing consumption of highly processed foods, and data related to colorectal cancer in the more developed regions of Brazil are comparable to those reported worldwide (2). Although recommended measures for the prevention of colorectal cancer (the use of the fecal occult blood test, flexible sigmoidoscopy, or colonoscopy) have been widely recognized and employed since 1993 (1), there have yet to be any HEEs of the use of such technologies in Brazil. The majority of studies of colorectal cancer in Brazil have focused on medications used in its treatment, which have been evaluated since 2008, including biologics such as the monoclonal antibody bevacizumab, approved by the FDA in 2004 and evaluated in an HEE related to Brazil in 2012 (49–52).

Consistent with these findings, another review of 242 cancer-related CUAs published in different parts of the world, through 2007, showed that the most frequent cancers studied were breast cancer (36% of studies), colorectal cancer (12%), and hematologic cancers (10%) (70, 71). In Spain, 13.0% of economic evaluations, published between 1990 and 2009, referred to cancer-related processes (72), and the disease most frequently evaluated was non-small cell lung cancer (31 %) (73).

Cancer treatment is a major focus of innovation in the field of medicine. Worldwide, the pharmacological treatment of cancer has an annual cost of approximately 40 billion US dollars (74). There have been a great number of studies showing that ICERs for oncological treatments, especially for new pharmacological therapies, are not below the threshold that would favor the incorporation of such treatments (6, 7). However, despite the general perception that the costs of treating cancer have increased, it should be borne in mind that technological innovations in the area of individualized medical treatment and the fact that the patents for many high-cost medications have lapsed present opportunities for reducing those costs (75).

Because the cost-effectiveness threshold for the incorporation of new technologies has yet to be defined in Brazil, the comparison of the ICERs estimated in oncology studies, using similar measures in different areas of medicine, could guide health care managers in their resource allocation decisions or at least inform them of which cancer treatments are the most costly. The WHO recommended, as a reference, that the threshold for the incorporation of a new technology into a health care system should be between one and three times the per capita GDP (12), but there is still no consensus on the subject (76). According to the Brazilian Institute of Geography and Statistics, the per capita GDP in Brazil, for 2013, was USD14,588.62. The ICERs reported for most of the health technologies evaluated in the HEEs under study were below the upper limit of the threshold suggested by the WHO. On the other hand, if the upper limit of York threshold were used (USD14,109.70), only 4 technologies would be considered cost-effective (77). However, of those same articles, 87.0% made recommendations in favor of incorporating the technologies evaluated, despite not having a cost-effectiveness threshold defined for Brazil and not even drawing comparisons with an international reference (such as that suggested by the WHO) or with technologies employed in other areas of medicine. This suggests a tendency, on the part of the scientific literature, to pressure health care systems to incorporate new technologies. Nevertheless, studies that addressed the relative value of oncologic pharmaceuticals used higher value thresholds and reported higher ICERs than studies evaluating noncancer drugs (78). Furthermore, because HTAs represent a relatively new research area in Brazil, it would not be surprising if most still do not rigorously adhere to international protocols and recommendations for the execution and publication of HEEs.

Only six technologies (five medications: Imatinib Mesylate, Rituximab, Erbitux, Trastuzumab, Gefitinib, Erlotinib, and one diagnostic test: PET-CT) evaluated in the HEE studies included in this review were analyzed by the Commission on Technology Incorporation—CITEC and National Committee for Health Technology Incorporation in SUS—CONITEC, advisory committees of the MoH on assignments relating to incorporation, exclusion or modification of health technologies by the SUS.

Our review has certain limitations that merit consideration. First, we did not evaluate the quality of the studies in accordance with established guidelines. Furthermore, it should be borne in mind that a considerable portion of the studies conducted in Brazil are supported by industries, and it is therefore possible that, in some cases, studies whose results were unfavorable to the incorporation of the technologies evaluated were not published. Coincidence or not, we noted that industry-supported studies tended to report lower ICERs, thus favoring the incorporation of the technologies evaluated (7).

Faced with budgetary constraints and rising health care costs, many countries have used HEEs as guides in making decisions regarding coverage, which has often limited patient access to costly new treatments (5). The lack of evidence from HEEs and unfavorable ICERs have both been used in order to justify the refusal to cover such treatments (7). Therefore, the explosion of high-cost innovations in oncology (71, 74) has created a common need to realign economic incentives, to improve communication at the clinical level, and to give greater weight to scientific evidence regarding the cost-effectiveness of cancer care (6, 7).

It is fundamental that growth in the area of HEEs related to Brazil be stimulated and that reviews of that literature be conducted in order to aggregate the data and allow the technologies employed in various areas of health care to be compared, in terms of their cost-effectiveness. Likewise, it is important to encourage discussions regarding the cost-effectiveness threshold in Brazil, which could serve as yet another objective criterion to inform and increase the transparency of the decision-making process surrounding the incorporation of new technologies into the standard practices of the SUS.

The incidence of cancer is on the rise worldwide, and the economic burden associated with its management has risen apace (1–3). Low- to middle-income countries, such as Brazil, will bear the brunt of that burden. One of the major challenges facing those countries is that of devising strategies to deal with their limited resources, allocating those resources appropriately in their management of cancer cases (79). Therefore, we hope that our findings will promote the incorporation of cost-effectiveness analyses in the decision-making processes regarding the coverage of and reimbursements for the use of cancer treatment technologies in Brazil.

## Conclusion

There has been an increase in the number of HEEs related to cancer in Brazil. The majority of the ICERs reported in such studies have been below the cost-effectiveness threshold suggested by the WHO for the incorporation of new health technologies. From a research standpoint, we emphasize the need to evaluate the quality of such studies conducted in Brazil.

The findings may contribute to support policymakers, researchers and clinicians in different ways. Policymakers can identify the most efficient technologies, which leads to a better allocation of resources. Researchers can visualize knowledge gaps to propose new HTA studies not yet carried out in the Brazilian context. Furthermore, clinicians can better visualize the high relation between costs and benefits, thus they can contribute to a more rational and responsible use of the technologies offered by the health system.

## Author contributions

All authors drafted the systematic review protocol. AC, TY, TD, RL, and PdS conducted the search, selection of records, and data extraction. Quality appraisal was conducted by TY, TD, RL, and PdS. All authors have read and approved the final manuscript.

### Conflict of interest statement

The authors declare that the research was conducted in the absence of any commercial or financial relationships that could be construed as a potential conflict of interest.

## Footnotes

^1^https://ww2.ibge.gov.br/home/estatistica/indicadores/precos/inpc_ipca/defaultseriesHist.shtm.
