# Supplementary material for: Health Economic Evaluations of Cancer in Brazil: A Systematic Review
Source: Front Public Health. 2018 Jul 27;6:205. doi: 10.3389/fpubh.2018.00205 (PMC6072849; doi:10.3389/fpubh.2018.00205)
Supplement: Supplementary file 1 [file Table_1.DOCX]

**Appendix 1**

**Medline (Pubmed) search strategy**

| #1 | "Costs and Cost Analysis"[Mesh] |
| --- | --- |
| #2 | "Economics, Hospital"[Mesh] |
| #3 | "Economics, Medical"[Mesh] |
| #4 | "Economics, Nursing"[Mesh] |
| #5 | "Economics, Pharmaceutical"[Mesh] |
| #6 | #1 OR #2 OR #3 OR #4 OR #5 |
| #7 | pharmacoeconomic*[Title/Abstract] |
| #8 | costminimization[Title/Abstract] |
| #9 | costeffectiveness[Title/Abstract] |
| #10 | costbenefit[Title/Abstract] |
| #11 | costutility[Title/Abstract] |
| #12 | cost of illness[Title/Abstract] |
| #13 | costconsequence[Title/Abstract] |
| #14 | healtheconomics[Title/Abstract] |
| #15 | #7 OR #8 OR #9 OR #10 OR #11 OR #12 OR #13 OR #14 |
| #16 | #6 OR #15 |
| #17 | letter[PublicationType] |
| #18 | editorial[PublicationType] |
| #19 | historicalarticle[PublicationType] |
| #20 | #17 OR #18 OR #19 |
| #21 | #16 NOT #20 |
| #22 | Brazil[MeSHTerms] |
| #23 | Brazil |
| #24 | brasil[Affiliation] |
| #25 | brazil* |
| #26 | brasil* |
| #27 | brasil[Title/Abstract] |
| #28 | brazil[Title/Abstract] |
| #29 | #22 OR #23 OR #24 OR #25 OR #26 OR #27 OR #28 |
| #30 | #21 AND #29 |

**Embase search strategy**

| 1 | ‘healtheconomics’ |
| --- | --- |
| 2 | ‘economicevaluation’/exp |
| 3 | ‘health care cost’/exp |
| 4 | ‘pharmacoeconomics’/exp |
| 5 | pharmacoeconomics$ |
| 6 | ‘cost benefit’:ti,ab. |
| 7 | ‘cost effectiveness’:ti,ab. |
| 8 | ‘cost minimization’:ti,ab. |
| 9 | ‘cost utility’:ti,ab. |
| 10 | ‘cost of illness’:ti,ab. |
| 11 | ‘cost consequence’:ti,ab. |
| 12 | or/1-11 |
| 13 | letter.it. |
| 14 | note.it. |
| 15 | editorial.it. |
| 16 | or/13-15 |
| 17 | 12 not 16 |
| 18 | ‘Brazil’/exp |
| 19 | brasi$:ti,ab. |
| 20 | brazi$:ti,ab. |
| 21 | brazil:ca. |
| 22 | or/18-21 |
| 23 | 17 and 22 |

**HTA and NHS EED search strategy**

HTA

| #1 | Brasil |
| --- | --- |
| #2 | Brazil |
| #3 | #1 OR #2 |
| #4 | Full publication record:ZDT |
| #5 | #3 AND #4 |
| #6 | IN HTA FROM 1980 TO 2012 |
| #7 | #5 AND #6 |

NHS EED

| #1 | Brasil |
| --- | --- |
| #2 | Brazil |
| #3 | #1 OR #2 |
| #4 | ((Economic evaluation:ZDT and Bibliographic:ZPS) |
| #5 | (Economic evaluation:ZDT and Abstract:ZPS)) |
| #6 | #4 OR #5 |
| #7 | #3 AND #6 |
| #8 | IN NHSEED FROM 1980 TO 2012 |
| #9 | #7 AND #8 |

**Scopus and Web of Science search strategy**

Scopus

| ((((((((((((("Costs and Cost Analysis")) OR pharmacoeconomic$) OR cost minimization) OR cost effectiveness) OR cost benefit) OR cost utility) OR cost of illness) OR cost consequence) OR health economics)) AND NOT (((letter) OR editorial) OR historical article))) AND ((("Brazil") OR ((brazil OR brasil))) OR ((brazil OR brazi$ OR brasi$ OR brasil))) AND (LIMIT-TO(AFFILCOUNTRY, "Brazil")) |
| --- |

Web of Science

| (((((((((((((("Costs and Cost Analysis")) OR pharmacoeconomic$) OR cost minimization) OR cost effectiveness) OR cost benefit) OR cost utility) OR cost of illness) OR cost consequence) OR health economics)) NOT (((letter) OR editorial) OR historical article))) AND ((("Brazil") OR ((brazil OR brasil))) OR ((brazil OR brazi$ OR brasi$ OR brasil)))) |
| --- |
